# Adjuvant Chemotherapy and Radiotherapy in Resected Pancreatic Ductal Adenocarcinoma: A Systematic Review and Clinical Practice Guideline

**DOI:** 10.3390/curroncol30070482

**Published:** 2023-07-08

**Authors:** James J. Biagi, Roxanne Cosby, Mala Bahl, Tarek Elfiki, Rachel Goodwin, Julie Hallet, Khalid Hirmiz, Aamer Mahmud

**Affiliations:** 1 Cancer Centre of Southeastern Ontario, 25 King Street West, Kingston, ON K7L 5P9, Canada; aamer.mahmud@kingstonhsc.ca; 2 Program in Evidence-Based Care, Department of Oncology, Juravinski Campus, McMaster University, 711 Concession Street, Hamilton, ON L8V 1C3, Canada; 3 Trillium Health Partners, 2200 Ellington Avenue West, Mississauga, ON L5M 2N1, Canada; mala.bahl@thp.ca; 4 Windsor Regional Cancer Centre, 2220 Kildare Road, Windsor, ON N8W 2X3, Canada; Tarek_elfiki@wrh.on.ca (T.E.); khalid.hirmiz@wrh.on.ca (K.H.); 5 The Ottawa Hospital Cancer Centre, 501 Smyth Road, Ottawa, ON K1H 8L6, Canada; rgoodwin@toh.on.ca; 6 Odette Cancer Centre, 2075 Bayview Avenue, Toronto, ON M4N 3M4, Canada; julie.hallet@sunnybrook.ca

**Keywords:** pancreatic ductal carcinoma, adjuvant treatment, chemoradiation therapy, stereotactic body radiation therapy

## Abstract

Pancreatic cancer is the seventh leading cause of cancer deaths worldwide, accounting for 4.7% of all cancer deaths, and is expected to climb significantly over the next decade. The purpose of this systematic review and guidance document was to synthesize the evidence surrounding the role of adjuvant treatment (chemotherapy and chemoradiation therapy [CRT], and stereotactic body radiation therapy [SBRT]) in resected pancreatic ductal adenocarcinoma (PDAC). Systematic literature searches of MEDLINE, EMBASE, and 11 guideline databases were conducted. Both direct and indirect comparisons indicate adjuvant chemotherapy offers a survival advantage over surgery alone. The optimal regimens recommended are mFOLFIRINOX with alternative options of gemcitabine plus capecitabine, gemcitabine alone, or S-1 (which is not available in North America). Trials comparing a CRT strategy to modern chemotherapy regimens are lacking. However, current evidence demonstrates that the addition of CRT to chemotherapy does not result in a survival advantage over chemotherapy alone and is therefore not recommended. Trials evaluating SBRT in PDAC are also lacking. SBRT should only be used within a clinical trial or multi-institutional registry.

## 1. Introduction

Pancreatic cancer is the eleventh most common cancer, accounting for a projected 2.9% of all new incident cases in 2022 in Canada. Despite this low incidence rate, pancreatic cancer is the third leading cause of cancer deaths in Canada, at 6.7% based on 2022 projections. Approximately 6900 Canadians will be diagnosed with pancreatic cancer in 2022, and 5600 will die from it [1]. Worldwide estimates for 2020 indicate that pancreatic cancer is the twelfth most common cancer and accounts for 2.7% of new incident cases and 4.7% of deaths [2]. Importantly, the projected increase in the incidence of pancreatic cancer over the next several years is expected to make pancreatic cancer the second most common cause of death by 2030 [3]. Surgery is the pillar of curative treatment for pancreatic cancer, followed by adjuvant chemotherapy [4]. Prior to recently published evidence of emerging chemotherapeutic regimens, the standard adjuvant chemotherapy consisted of a six-month course of either gemcitabine or a 5-FU/leucovorin regimen [5,6,7]. However, in the context of recently published evidence of these emerging regimens, the optimal regimen to use has been unclear. Moreover, there remain questions regarding the utility of including adjuvant radiation in adjuvant chemotherapy as well as questions surrounding the use of adjuvant SBRT, which is a newer technology.

The purpose of this guidance document was to synthesize the evidence surrounding the role of adjuvant treatment (chemotherapy, CRT, and SBRT) in resected PDAC, as outlined in the research questions below. To our knowledge, this manuscript is the most up-to-date systematic review that incorporates the results of the most recent randomized trials, including ACCORD, ESPAC-4, and APACT.

This systematic review was conducted according to PRISMA guidelines; however, it was not registered with PRISMA.

This systematic review has been registered on the PROSPERO website with registration number CRD42020179816 (https://www.crd.york.ac.uk/PROSPERO/#recordDetails [accessed on 29 March 2023]).

## 2. Research Questions

- What is the contemporary role of adjuvant chemotherapy in the treatment of patients with resected pancreatic ductal adenocarcinoma (PDAC) with respect to overall survival (OS), progression-free survival (PFS), toxicity/safety, and quality of life (QOL)?
- What is the role of adjuvant chemoradiation therapy (CRT) in the treatment of patients with resected PDAC with respect to OS, PFS, toxicity/safety, and QOL?
- What is the role of adjuvant stereotactic body radiation therapy (SBRT) in the treatment of patients with resected PDAC with respect to OS, PFS, toxicity/safety, and QOL?

## 3. Methods

The PEBC uses the methods of the Practice Guidelines Development Cycle [8,9] produce evidence-based and evidence-informed guidelines. This is an established process that includes a systematic review and interpretation of the evidence by a Working Group of content experts who formulate draft recommendations. The draft recommendations are vetted through an internal review process by content and methodology experts, which is followed by an external review process by Ontario clinicians and other stakeholders.

The PEBC also uses the AGREE II framework [10] as a methodological strategy for guideline development. AGREE II is a well-validated tool designed to assess the methodological rigor and transparency of the development of guidelines. It is also designed to improve the completeness and transparency of the reporting in the guidelines.

This systematic review was conducted according to PRISMA guidelines (http://www.prisma-statement.org/?AspxAutoDetectCookieSupport=1 (accessed on 29 March 2023)); however, it was not registered with PRISMA (see Supplemental Table S1).

The project was led by a small working group drawn from within the Gastrointestinal Disease Site Group. This group had expertise in medical oncology, radiation oncology, surgical oncology, and health research methodology.

This systematic review of the evidence was conducted in three planned stages: a search for guidelines, systematic reviews, and primary literature. These stages are briefly described in subsequent sections below. For a full description of the methods used in the development of this systematic review and clinical practice guideline, please refer to the full guidance document found at https://www.cancercareontario.ca/en/guidelines-advice/types-of-cancer/71976 (accessed on 1 June 2023).

### 3.1. Literature Searches

#### 3.1.1. Search for Guidelines

A search for existing evidence-based guidelines was undertaken to determine whether there was a suitable existing guideline that could be endorsed for any of the research questions. Relevant guidelines were evaluated for quality using the AGREE II instrument [10]. The criterion for endorsement was that the AGREE II rigor of the development domain, which assesses the methodological quality of the guideline, was above 50%.

No suitable guidelines were found in these sources. MEDLINE and EMBASE were also searched. The search strategy can be obtained from the corresponding author upon request.

#### 3.1.2. Search for Systematic Reviews

Current systematic reviews were searched for in the MEDLINE and EMBASE databases from 2016 to November 25,2021. Inclusion criteria were English language systematic reviews that covered any of the current guideline questions with similar inclusion/exclusion criteria that did not have an existing evidence-based guideline to endorse or adapt.

Identified systematic reviews were assessed using the AMSTAR 2 [11] tool to determine whether existing systematic reviews met a minimum threshold for methodological quality and could be considered for inclusion in the evidence base.

#### 3.1.3. Search for Primary Literature

If no guideline or systematic review was identified for a particular research question, then a search for primary literature was conducted. Moreover, an updated search for primary literature was performed for any included systematic review from the point in time that the existing systematic review search ended.

MEDLINE and EMBASE were searched for primary studies beginning in January 2000 if there was no systematic review included for a given question. If a systematic review was included, the search for primary studies began from the point that the search timeframe from the included systematic review ended.

 
*Inclusion Criteria*

- English language
- Adults with resected PDAC
- Included a comparison of interest as per the guideline questions.
- Included at least one outcome of interest: OS, PFS, toxicity/safety, QOL.
- Randomized Controlled Trials (RCTs) (if available). If RCTs were not available, other comparative studies were retained.
- N = 30 minimally

A review of the titles and abstracts was conducted independently by one reviewer (RC). For studies that warranted full-text review, one reviewer (RC) reviewed each study. If uncertainty existed for a given study, a second reviewer (JB) would independently review the paper in question, and a consensus on applicability would be reached.

### 3.2. Data Extraction and Assessment of Risk of Bias

All included primary studies underwent data extraction by one GDG member (RC), with all extracted data and information audited subsequently by an independent auditor.

Cochrane Risk of Bias tool (RoB2) was used to assess the potential bias of all included RCTs (https://sites.google.com/site/riskofbiastool/ (accessed on 29 March 2023)).

### 3.3. Synthesizing the Evidence

Meta-analysis was not planned owing to the use of existing systematic reviews with meta-analyses and existing guidelines.

### 3.4. Assessment of the Certainty of the Evidence

The certainty of the evidence, considering the risk of bias, inconsistency, indirectness, imprecision, and publication bias, was assessed for each of the research questions.

### 3.5. Internal Review

For the guideline document to be approved, 75% of the Internal Review Expert Panel had to cast a vote, and of those who voted, 75% had to approve the document. In addition, a PEBC three-person Report Approval Panel must unanimously approve the document. Members of either panel could request changes to the document.

A consultation group consisting of four patients/survivors/caregivers also reviewed the project plan and draft recommendations at the appropriate times and provided feedback on their comprehensibility, appropriateness, and feasibility.

### 3.6. External Review

The External Review consisted of two processes. The first was Targeted Peer Review, where several content experts were asked to review and provide feedback on the guidance document. The second process was Professional Consultation, where end-users of the guidance document are sent an online survey and asked to provide feedback on the guideline recommendations.

## 4. Results

### 4.1. Literature Searches

#### 4.1.1. Search for Guidelines

A guideline search uncovered 832 guidelines, of which 43 underwent a full-text review. One guideline by Palta et al. [12] was retained as an appropriate source document for endorsement for Question 3 only. Two reviewers evaluated this guideline independently using the AGREE II tool. The rigor of the development domain was 81%, which met the a priori endorsement criterion noted above. All other domains were between 78% and 96% except the applicability domain, which scored 40%. This was mainly owing to this guideline not discussing the potential resource implications of applying their recommendations. Overall, both reviewers recommended this guideline for use. AGREE II evaluations are available in Supplemental Table S2.

#### 4.1.2. Search for Systematic Reviews

**Figure 1:**
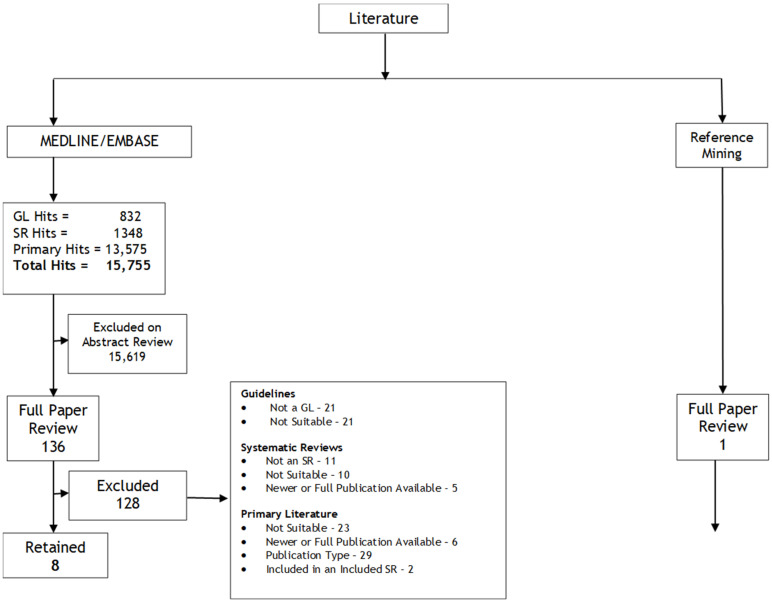
Literature search results flow diagram.

A search for systematic reviews uncovered 1348 documents. Of these, 29 underwent full-text review, and three [13,14,15] met the pre-planned inclusion criteria (Figure 1). These systematic reviews were used for Questions 1 and 2.

#### 4.1.3. Search for Primary Literature

A relevant systematic review was identified for Questions 1 and 2. Therefore, the search for primary literature was conducted beginning from the point where the searches for the included systematic reviews ended. A search for primary literature for Question 3 was not necessary as a recommendation from an existing guideline was adopted.

For the individual study literature search, there were 13,575 hits. Of these, 64 underwent a full-text review, and four were retained: RTOG 0848 [16], as well as the Taiwan Cooperative Oncology Group (TCOG) T3207 trial [17], an update of the APACT trial [18], and an update of the ESPAC-4 trial [19], which were in abstract form. A search for a full publication of the TCOG T3207 trial yielded one other abstract [20]. In addition, a search of the reference lists of all included studies yielded an additional study for inclusion. A summary of the full literature search is found in Figure 1.

### 4.2. Certainty of the Evidence

Various study designs are included in this guidance document. The included guideline was evaluated using the AGREE II tool [10] and was deemed to be of sufficient quality to be included in the current guidance document. Three systematic reviews were retained and evaluated using the AMSTAR 2 tool [11]. RCTs were assessed using the second version of the Cochrane Risk of Bias tool (RoB2) (https://sites.google.com/site/riskofbiastool/ (accessed on 29 March 2023)) (Table 1).

Three systematic reviews [13,14,15] were retained and were evaluated using the AMSTAR 2 tool [11]. The evaluations are available in Supplemental Table S3. These systematic reviews only included RCTs, and overall, they were strong methodologically, having scored a ‘yes’ to most of the items included in this tool. There were only a few items that were scored a ‘no’ for each study. No systematic review provided a list of excluded studies; however, this is understandable as these lists would have numbered in the thousands. No systematic review provided the sources of funding for the studies included in the reviews. It is unknown if these data were not collected or if they were just not reported for brevity. No systematic review included an evaluation of publication bias. The results in all these systematic reviews suffer from indirectness owing to the differences in chemotherapy regimens used in each of the included studies.

Four RCTs presented in five publications [16,17,18,19,20] were included in this guidance document and were assessed using Cochrane’s RoB2 (chapter 8) (https://sites.google.com/site/riskofbiastool/ (accessed on 29 March 2023)) (Table 1). The trial by Abrams et al. [16] was assessed to have a low risk of bias in all domains of the RoB2 and therefore had an overall low risk of bias for all outcomes. All other trials had at least one domain with some concerns. The TCOG T3207 trial was only reported in abstract form [17,20], which makes it difficult to assess the true risk of bias owing to the limited information available.

### 4.3. Outcomes

#### 4.3.1. What Is the Contemporary Role of Adjuvant Chemotherapy in the Treatment of Patients with Resected PDAC?

Two systematic reviews with network meta-analyses were retained [13,14]. The Parmar et al. [13] systematic review included phase III trials of adjuvant chemotherapy in resected PDAC. Direct pairwise random effects meta-analysis was conducted where possible. Indirect comparisons were evaluated using network meta-analysis. This systematic review was comprised of 10 publications of 11 important RCTs that included 4920 participants (two RCTs were reported in one paper). Five RCTs compared adjuvant chemotherapy to observation: CONKO-001 [21], JSAP-02 [22], ESPAC-3 (v1) [23], ESPAC-1 Plus [23], and ESPAC-1 [5]. Six RCTs compared two different adjuvant chemotherapy regimens: APACT [24], PRODIGE [25], ESPAC-4 [26], CONKO-005 [27], JASPAC-01 [28], and ESPAC-3 [6]. In all but one of the trials, 55% to 88% of participants had R0 resections. ESPAC-4 [26] was the lone exception, wherein 60% of participants had an R1 resection.

Results of direct pairwise meta-analysis comparing adjuvant chemotherapy with observation demonstrated significantly better overall disease-free survival (DFS) (HR, 0.56; 95% CI, 0.46 to 0.68; *p* < 0.00001) with adjuvant chemotherapy. Likewise, direct pairwise meta-analysis comparing other adjuvant chemotherapy regimens with gemcitabine alone also demonstrated significantly better DFS (HR, 0.76; 95% CI, 0.63 to 0.92, *p* = 0.005) for other adjuvant chemotherapy. Indirect comparisons using network meta-analysis demonstrated that DFS was significantly improved with modified FOLFIRINOX (mFOLFIRINOX) compared with 5-FU (HR, 0.56; 95% CI, 0.43 to 0.73), gemcitabine (HR, 0.58; 95% CI, 0.46 to 0.73), gemcitabine plus capecitabine (HR, 0.67; 95% CI, 0.51 to 0.90), gemcitabine plus erlotinib (HR, 0.62; 95% CI, 0.453 to 0.84), and gemcitabine plus nab-paclitaxel (HR, 0.66; 95% CI, 0.49 to 0.89). Similar improvements in DFS were also reported with S-1 compared with 5-FU, gemcitabine, gemcitabine plus capecitabine, gemcitabine plus erlotinib, and gemcitabine plus nab-paclitaxel [13].

Results of direct pairwise meta-analysis comparing adjuvant chemotherapy with observation demonstrated significantly better OS (HR, 0.73; 95% CI, 0.63 to 0.84; *p* < 0.00001) with adjuvant chemotherapy. Similarly, direct pairwise meta-analysis comparing other adjuvant chemotherapy regimens with gemcitabine alone also demonstrated significantly better OS (HR, 0.72; 95% CI, 0.61 to 0.86, *p* = 0.0002) for other adjuvant chemotherapy. Indirect comparisons using network meta-analysis demonstrated that OS was significantly improved with mFOLFIRINOX compared with 5-FU (HR, 0.64; 95% CI, 0.46 to 0.90), gemcitabine (HR, 0.64; 95% CI, 0.47 to 0.87), and gemcitabine plus erlotinib (HR, 0.68; 95% CI, 0.47 to 1.00), but not gemcitabine plus capecitabine (HR, 0.78; 95% CI, 0.54 to 1.12) or gemcitabine plus nab-paclitaxel (HR, 0.78; 95% CI, 0.54 to 1.13). Likewise, indirect comparisons using network meta-analysis demonstrated that OS was significantly improved with S-1 compared with 5-FU, gemcitabine, gemcitabine plus capecitabine, gemcitabine plus erlotinib, and gemcitabine plus nab-paclitaxel [13].

Updated OS results for APACT [18] and ESPAC-4 [19] are similar to the results initially reported for these trials that were included in the Parmar et al. [13] network meta-analysis.

Results of direct pairwise comparisons of grade 3/4 hematological toxicities demonstrate no significant differences between other adjuvant chemotherapy and gemcitabine with respect to thrombocytopenia (HR, 0.64; 95% CI, 0.27 to 1.50; *p* = 0.30), neutropenia (HR, 0.85; 95% CI, 0.55 to 1.32; *p* = 0.48), and febrile neutropenia (HR, 1.27, 95% CI, 0.48 to 3.38; *p* = 0.63). However, anemia was significantly improved with other adjuvant chemotherapy compared with gemcitabine alone (HR, 0.74; 95% CI, 0.59 to 0.94; *p* = 0.01) [4]. No QOL data were reported [13].

The Kamarajah et al. [14] network meta-analysis was very similar to the Parmar et al. [13] network meta-analysis, covering almost the exact same time frame. The same conclusion, that the optimal adjuvant chemotherapy following resection for PDAC is S-1 or mFOLFIRINOX, was reported.

A search for primary studies from the point that the Parmar et al. [13] systematic review ended yielded one publication of a randomized phase II trial comparing gemcitabine to gemcitabine plus erlotinib in those with resected head of pancreas adenocarcinoma [16]. Median OS for gemcitabine plus erlotinib versus gemcitabine was 28.1 months versus 29.9 months (HR, 1.04; 95% CI, 0.79 to 1.38; *p* = 0.62 [one-sided, log-rank]). Moreover, there was no DFS advantage to the combination chemotherapy regimen compared with gemcitabine monotherapy (HR, 1.02; 95% CI, 0.80 to 1.31; *p* = 0.58 [one-sided, log-rank]).

The positive results from Japanese JASPAC-01 [28] were included in both the Kamarajah et al. [14] and Parmar et al. [13] analyses. However, the applicability to European and North American patients has not been established. Pharmacokinetics and pharmacodynamics of S-1 differences may account for the increased toxicities in the latter populations. We have therefore not included studies that used S-1 in our analysis or recommendation development.

Overall, mFOLFIRINOX is an appropriate adjuvant chemotherapy for fit patients. In patients for whom mFOLFIRINOX is not suitable, gemcitabine plus capecitabine or gemcitabine alone are appropriate options.

#### 4.3.2. What Is the Role of Adjuvant CRT in the Treatment of Patients with Resected PDAC?

One systematic review with network meta-analysis was retained [15]. The Xu et al. [15] study was designed to determine the optimal adjuvant chemotherapy for resected pancreatic adenocarcinoma. A total of 13 RCTs that included 4098 participants were included in the network meta-analysis; however, for the purposes of the current guideline, only the trial data dealing with CRT were considered: Regine et al. [29], EORTC 40,891 [30], ESPAC-1 [5], and Kalser et al. [31]. There was no significant difference between fluorouracil and fluorouracil plus CRT with respect to one-year survival (HR, 1.07; 95% CI, 0.44 to 2.53), three-year survival (HR, 1.28; 95% CI, 0.64 to 2.46), and five-year survival (HR, 1.88; 95% CI, 0.60 to 7.02). Likewise, there was no significant difference between gemcitabine and gemcitabine plus CRT with respect to one-year survival (HR, 0.86; 95% CI, 0.20 to 3.59), three-year survival (HR, 0.93; 95% CI, 0.33 to 2.57), and five-year survival (HR, 1.77; 95% CI, 0.30 to 11.98). Although gemcitabine plus CRT resulted in more toxicity than gemcitabine alone, the difference was not statistically significant (HR, 0.70; 95% CI, 0.00 to 537.3). No QOL data were reported.

A search for primary studies from the point that the Xu et al. [15] systematic review ended yielded two abstracts of one RCT [17,20]. The TCOG T3207 trial (147 participants) compared adjuvant gemcitabine with adjuvant gemcitabine plus CRT. Although the 2019 ESMO abstract was the more recent publication, the 2018 ESMO abstract contained much more information; therefore, it was also included. The primary endpoint was recurrence-free survival. There was no significant difference in median RFS in the two arms of this trial (12.1 months vs. 13.3 months; HR, 0.96; 95% CI, 0.67 to 1.37; *p* = 0.80) or in OS (23.5 months vs. 21.5 months; HR, 1.07; 95% CI, 0.74 to 1.55; *p* = 0.73). Moreover, grade 3/4 toxicity was similar in the two arms (66% vs. 73%, *p* = 0.34).

Current evidence regarding the use of adjuvant chemoradiation is insufficient to support its routine use in those who have resected PDAC.

#### 4.3.3. What Is the Role of Adjuvant SBRT in the Treatment of Patients with Resected PDAC?

SBRT is capable of delivering high doses of radiation to the tumor precisely, provided the volume is not too large. This mode of delivering radiation therapy involves motion management while attempting to minimize the risk of treatment-related toxicities to adjacent organs. SBRT continues to be explored in terms of treatment fields, dose, technical factors, toxicities, and the impact on outcomes.

One guideline produced by the American Society for Radiation Oncology (ASTRO) [12] was retained from the guideline search as it sufficiently addressed the issue of SBRT following resection of PDAC and was therefore endorsed by the Working Group. Only the recommendation pertaining to adjuvant SBRT is being endorsed (see page 326 of the Palta et al. [12] guideline). The authors of this guideline conducted a systematic review and recommend that adjuvant SBRT only be used within a clinical trial or multi-institutional registry. This recommendation was considered strong, although the quality of the evidence it was based on was very low. All members of the ASTRO guideline Working Group agreed with this recommendation.

The role of radiation in the management of pancreatic cancer is evolving, as is the use of SBRT, with advances in motion management, target delineation, treatment planning, and image guidance. Such techniques have furthered dose escalation and ablative RT with improved local control and quality of life and acceptable toxicity. RT is likely to become even more important as new systemic therapies are developed and there is increased focus on controlling local disease. The appropriate role and integration of SBRT with systemic therapy and surgery is an ongoing area of active investigation [12].

## 5. Discussion

Comparative adjuvant chemotherapy trials have led to a shift from single-agent chemotherapy to more effective combination regimens. Gemcitabine monotherapy has been the control arm of choice in several large-scale randomized trials reported. The PRODIGE [25] and ESPAC-4 [26] trials with experimental arms of mFOLFIRINOX and gemcitabine plus capecitabine, respectively, met their study endpoints and have been adopted as new standards. Unfortunately, the APACT trial [24] of gemcitabine plus nab-paclitaxel was interpreted as a negative study, despite the established role of this regimen in the first-line metastatic setting.

The recommendations in Question 1 stating a preference in favor of the mFOLFIRINOX regimen versus the gemcitabine plus capecitabine or gemcitabine-alone regimens are based on the comparative survival advantage derived in the network meta-analysis [13]. An important qualification here is that there is a lack of direct comparative data for efficacy or toxicities. Additionally, important patient characteristics, including R1 resection, nodal status, and baseline CA19-9 varied considerably among the trials. Thus, it is important at a practical level that patient characteristics, perhaps the most important being functional status, influence the choice of adjuvant regimen when the discussion between caregiver and patient takes place.

The role of radiation in the adjuvant setting continues to be debated. Trials comparing a radiation plus chemotherapy (CRT) strategy to modern chemotherapy regimens are lacking. ESPAC-1 trial showed no survival difference among 175 patients who received postoperative CRT when compared to the 178 who did not (median overall survival 15.5 versus 16.1 months, respectively) [32]. In the subsequent intent-to-treat analysis of the 289 patients, there was a trend toward worse survival for the group receiving CRT [5]. A meta-analysis of nine randomized trials comparing six different adjuvant strategies reflected a lack of precision, and it is difficult to draw any meaningful conclusions in terms of the benefit of CRT [33]. The EORTC 40013 phase II study randomly assigned 90 patients with resected pancreatic cancer (70% node positive) to two cycles of weekly gemcitabine alone followed by radiation therapy 5040 cGy in 28 daily fractions of 180 cGy. Initially, the control group was observation alone (*n* = 4). However, the protocol was amended, such that the remainder of the control group (*n* = 41) received four cycles of gemcitabine alone. The median overall survival was 24 months in both arms, and the DFS was 12 months versus 11 months in the control group. The rate of local recurrence in the CRT group was lower (11% vs. 24%), but the rates of distant progression were similar (40% vs. 42%) [34]. Additional information from NRG/RTOG 0848 on the role of CRT is awaited [16]. Based on the above data, many clinicians do not recommend concomitant CRT after resection of pancreatic cancer. However, some find adjuvant CRT a reasonable approach for patients who have high-risk features such as a positive margin (R1 resection) or node-positive disease and for whom FOLFIRINOX was not received. Evidence for this approach remains limited to subgroup analyses of larger trials. The conclusions in this guideline are consistent with statements from other specialty organizations, including ASCO [35] and ESMO [36].

An important trial comparing the fluoropyrimidine analogue S-1 to gemcitabine in the adjuvant setting demonstrated positive results in a Japanese population [14]. However, the applicability to European and North American patients has not been established. Pharmacokinetics and pharmacodynamics of S-1 differences may account for the increased toxicities in the latter populations. We have therefore not included studies that used S-1 in our analysis or recommendation development.

Among the varied areas of active clinical research in resectable PDAC, there are two current research priorities we wish to highlight. The neo-adjuvant setting is receiving a great deal of attention, as represented, for instance, by the US-led ALLIANCE A021806 and Dutch PREOPANC-3 trials. Patients with pancreatic cancer who have underlying germline mutations represent specific populations for which novel therapeutic approaches are under active investigation. Emerging evidence of agents that target such mutations in the advanced cancer setting, for example, poly adenosine diphosphate-ribose polymerase (PARP) inhibition for DNA damage repair mutations [37], may provide future options for patients in the adjuvant setting. In terms of the limitations of this guidance document, the literature with respect to the use of adjuvant CRT, particularly in combination with modern chemotherapy regimens, is limited in the setting of resected PDAC. The literature regarding adjuvant SBRT is also currently quite limited in this population. Further research in these areas is encouraged.

## 6. Guideline Recommendations

The following recommendations are made:

Adjuvant chemotherapy is recommended for patients with R0 or R1 resected pancreatic ductal adenocarcinoma. Modified FOLFIRNOX (mFOLFIRINOX) is recommended for appropriately fit patients. If a patient is not suitable for mFOLFIRINOX, alternative options include gemcitabine plus capecitabine or gemcitabine alone.

There is insufficient evidence to support the routine use of adjuvant chemoradiation for patients with R0 or R1 resected PDAC. The role of adjuvant CRT remains uncertain.

Following surgical resection of pancreatic cancer, adjuvant SBRT is only recommended on a clinical trial or multi-institutional registry (endorsed by the American Society for Radiation Oncology [ASTRO] guideline by Palta et al. [12]).

## Figures and Tables

**Table 1:** Evaluation of included randomized controlled trials using Cochrane’s Risk of Bias tool.

Study	Comparison	Randomization Bias	Deviations from the Intended Interventions Bias	Missing Outcome Data Bias	Measurement of Outcome Bias	Reporting Bias	Overall Risk of Bias
Tempero et al., 2021 [18] (APACT update) *abstract*	Nab-P/Gem vs. Gem	Low	Low	Low	Some concerns	Low	Some concerns
Neoptolemos et al., 2020 [19] (ESPAC-4 update) *abstract*	GemCap vs. Gem	Low	Low	Low	Some concerns	Low	Some concerns
Abrams et al., 2020 [16]	Gem vs. Gem/Erlotinib	Low	Low	Low	Low	Low	Low
TCOG T3207, 2018/19 [17,20] *abstract*	Gem vs. Gem/CRT	Some concerns	Some concerns	Low	Low	Some concerns	Some concerns

Abbreviations: CAP = capecitabine; CRT = chemoradiation; Gem = gemcitabine; nab-P = nab-paclitaxel.

## Data Availability

All data, analytic methods and study materials will be made available upon request to the corresponding author.

## Supplementary Materials

The following supporting information can be downloaded at: https://www.mdpi.com/article/10.3390/curroncol30070482/s1, Table S1: PRISMA 2020 Checklist; Table S2: Evaluation of included guideline using AGREE II; Table S3: Evaluation of included systematic reviews using AMSTAR2.

## Acronyms

Acronyms used in this guidance document:

AGREE	Appraisal of Guidelines, REsearch and Evaluation tool
AMSTAR	A MeaSurement Tool to Assess systematic Reviews
ASCO	American Society of Clinical Oncology
ASTRO	American Society for Radiation Oncology
cGY	centigray
CI	confidence interval
CRT	chemoradiation therapy
DFS	disease free survival
ECRI	Emergency Care Research Institute
ESMO	European Society for Medical Oncology
HR	hazard radio
OS	overall survival
*p*	*p* value
PDAC	pancreatic ductal adenocarcinoma
PFS	progression free survival
QOL	quality of life
RCT	randomized controlled trial
RoB	risk of bias
ROBINS-I	Risk Of Bias In Non-Randomized Studies-of Interventions tool
SBRT	stereotactic body radiation therapy
