# Supplementary material for: Adjuvant Chemotherapy and Radiotherapy in Resected Pancreatic Ductal Adenocarcinoma: A Systematic Review and Clinical Practice Guideline
Source: Curr Oncol. 2023 Jul 8;30(7):6575–86. doi: 10.3390/curroncol30070482 (PMC10378160; doi:10.3390/curroncol30070482)
Supplement: Supplementary file 1 [file curroncol-30-00482-s001.zip › curroncol-2382727-supplementary.pdf]

Table S1: PRISMA 2020 Checklist

Section and Topic	Item #	Checklist item	Location where item is reported
**TITLE**			
Title	1	Identify the report as a systematic review.	Title, Abstract, Methods (Section 3)
**ABSTRACT**			
Abstract	2	See the PRISMA 2020 for Abstracts checklist.	Abstract
**INTRODUCTION**			
Rationale	3	Describe the rationale for the review in the context of existing knowledge.	Section 1
Objectives	4	Provide an explicit statement of the objective(s) or question(s) the review addresses.	Section 2
**METHODS**			
Eligibility criteria	5	Specify the inclusion and exclusion criteria for the review and how studies were grouped for the syntheses.	Section 3
Information sources	6	Specify all databases, registers, websites, organisations, reference lists and other sources searched or consulted to identify studies. Specify the date when each source was last searched or consulted.	Section 3 – link to full methods in the original guidance document
Search strategy	7	Present the full search strategies for all databases, registers and websites, including any filters and limits used.	Section 3 – statement that search strategies available upon request to corresponding

Section and Topic	Item #	Checklist item	Location where item is reported
			author
Selection process	8	Specify the methods used to decide whether a study met the inclusion criteria of the review, including how many reviewers screened each record and each report retrieved, whether they worked independently, and if applicable, details of automation tools used in the process.	Section 3.1.3
Data collection process	9	Specify the methods used to collect data from reports, including how many reviewers collected data from each report, whether they worked independently, any processes for obtaining or confirming data from study investigators, and if applicable, details of automation tools used in the process.	Section 3.2
Data items	10a	List and define all outcomes for which data were sought. Specify whether all results that were compatible with each outcome domain in each study were sought (e.g. for all measures, time points, analyses), and if not, the methods used to decide which results to collect.	Section 4.3
	10b	List and define all other variables for which data were sought (e.g. participant and intervention characteristics, funding sources). Describe any assumptions made about any missing or unclear information.	NA
Study risk of bias assessment	11	Specify the methods used to assess risk of bias in the included studies, including details of the tool(s) used, how many reviewers assessed each study and whether they worked independently, and if applicable, details of automation tools used in the process.	Section 3.2, Table 1
Effect measures	12	Specify for each outcome the effect measure(s) (e.g. risk ratio, mean difference) used in the synthesis or presentation of results.	Section 4.3
Synthesis methods	13a	Describe the processes used to decide which studies were eligible for each synthesis (e.g. tabulating the study intervention characteristics and comparing against the planned groups for each synthesis (item #5)).	Section 3.2
	13b	Describe any methods required to prepare the data for presentation or synthesis, such as handling of missing summary statistics, or data conversions.	NA
	13c	Describe any methods used to tabulate or visually display results of individual studies and syntheses.	NA
	13d	Describe any methods used to synthesize results and provide a rationale for the choice(s). If meta-analysis was performed, describe the model(s), method(s) to identify the presence and extent of statistical heterogeneity, and software package(s) used.	Section 3.3
	13e	Describe any methods used to explore possible causes of heterogeneity among study results (e.g. subgroup analysis, meta-regression).	NA (no meta-analysis)
	13f	Describe any sensitivity analyses conducted to assess robustness of the synthesized results.	NA

Section and Topic	Item #	Checklist item	Location where item is reported
Reporting bias assessment	14	Describe any methods used to assess risk of bias due to missing results in a synthesis (arising from reporting biases).	NA
Certainty assessment	15	Describe any methods used to assess certainty (or confidence) in the body of evidence for an outcome.	Section 3.4
**RESULTS**			
Study selection	16a	Describe the results of the search and selection process, from the number of records identified in the search to the number of studies included in the review, ideally using a flow diagram.	Section 4.1, Figure 1
	16b	Cite studies that might appear to meet the inclusion criteria, but which were excluded, and explain why they were excluded.	NA
Study characteristics	17	Cite each included study and present its characteristics.	Section 4.1
Risk of bias in studies	18	Present assessments of risk of bias for each included study.	Section 3.2, Table 1
Results of individual studies	19	For all outcomes, present, for each study: (a) summary statistics for each group (where appropriate) and (b) an effect estimate and its precision (e.g. confidence/credible interval), ideally using structured tables or plots.	Section 4.3
Results of syntheses	20a	For each synthesis, briefly summarise the characteristics and risk of bias among contributing studies.	Section 3.2, Table 1
	20b	Present results of all statistical syntheses conducted. If meta-analysis was done, present for each the summary estimate and its precision (e.g. confidence/credible interval) and measures of statistical heterogeneity. If comparing groups, describe the direction of the effect.	Section 4.3
	20c	Present results of all investigations of possible causes of heterogeneity among study results.	NA (no meta-analysis)
	20d	Present results of all sensitivity analyses conducted to assess the robustness of the synthesized results.	NA
Reporting biases	21	Present assessments of risk of bias due to missing results (arising from reporting biases) for each synthesis assessed.	NA
Certainty of	22	Present assessments of certainty (or confidence) in the body of evidence for each outcome assessed.	Section 4.2

Section and Topic	Item #	Checklist item	Location where item is reported
evidence			
**DISCUSSION**			
Discussion	23a	Provide a general interpretation of the results in the context of other evidence.	Section 5
	23b	Discuss any limitations of the evidence included in the review.	Section 5
	23c	Discuss any limitations of the review processes used.	Section 5
	23d	Discuss implications of the results for practice, policy, and future research.	Section 5
**OTHER INFORMATION**			
Registration and protocol	24a	Provide registration information for the review, including register name and registration number, or state that the review was not registered.	Section 1
	24b	Indicate where the review protocol can be accessed, or state that a protocol was not prepared.	Data Availability Statement
	24c	Describe and explain any amendments to information provided at registration or in the protocol.	NA
Support	25	Describe sources of financial or non-financial support for the review, and the role of the funders or sponsors in the review.	Funding Statement
Competing interests	26	Declare any competing interests of review authors.	Conflict of Interest Statement
Availability of data, code and other materials	27	Report which of the following are publicly available and where they can be found: template data collection forms; data extracted from included studies; data used for all analyses; analytic code; any other materials used in the review.	Data Availability Statement

From: Page MJ, McKenzie JE, Bossuyt PM, Boutron I, Hoffmann TC, Mulrow CD, et al. The PRISMA 2020 statement: an updated guideline for reporting systematic reviews. BMJ 2021;372:n71. doi: 10.1136/bmj.n71For more information, visit: http://www.prisma-statement.org/

Table S2: Evaluation of included guideline using AGREE II.

DOMAIN	ITEM	APPRAISER 1	APPRAISER 2	DOMAIN SCORE
Scope and Purpose	1. The overall objective of the guideline is (are) specifically described.	7	6	89%
	2. The health question(s) covered by the guideline is (are) specifically described.	7	7	
	3. The population to whom the guideline is meant to apply is specifically described.	6	5	
Stakeholder Involvement	4. The guideline development group includes individuals from all the relevant professional groups.	7	6	78%
	5. The views and preferences of the target population have been sought.	7	5	
	6. The target users of the guideline are clearly defined.	5	4	
Rigour of Development	7. Systematic methods were used to search for evidence.	6	6	81%
	8. The criteria for selecting the evidence are clearly described.	5	4	
	9. The strengths and limitations of the body of evidence are clearly described.	5	5	
	10. The methods for formulating the recommendation are clearly described.	5	5	
	11. The health benefits, side effects and risks have been considered in formulating the recommendations.	6	6	
	12. There is an explicit link between the recommendations and the supporting evidence.	7	7	
	13. The guideline has been externally reviewed by experts prior to its publication.	7	6	
	14. A procedure for update the guideline is provided.	7	7	
Clarity of Presentation	15. The recommendations are specific and unambiguous.	7	7	92%
	16. The different options for management of the condition or health issue are clearly presented.	6	6	
	17. Key recommendations are easily identifiable.	7	6	
Applicability	18. The guideline describes facilitators and barriers to its application.	4	4	40%
	19. The guideline provides advice and/or tools on how the recommendations can be put into practice.	4	4	

	20. The potential resource implications of applying the recommendations have been considered.	1	1	
	21. The guideline presents monitoring and/or auditing criterial.	4	5	
Editorial Independence	22. The views of the funding body have not influenced the content of the guideline.	7	6	96%
	23. Competing interests of guideline development group members have been recorded and addressed.	7	7	
Overall Guideline Assessment	Rate the overall quality of this guideline.	6	6	
	I would recommend this guideline for use.	Yes	Yes	

Table S3. Evaluation of included systematic reviews using AMSTAR2.

**ITEM**	**Pa rm**	**Ka m**	**Xu et**
**1.** Did the research questions and inclusion criteria for the review include the components of PICO?	Y	Y	Y
**2.** Did the report of the review contain an explicit statement that the review methods were established prior to the conduct of the review and did the report justify any significant deviations from the protocol?	PY	Y	Y
**3.** Did the review authors explain their selection of the study designs for inclusion in the review?	Y	Y	Y
**4.** Did the review authors use a comprehensive literature search strategy?	Y	Y	Y
**5.** Did the review authors perform study selection in duplicate?	Y	Y	N
**6.** Did the review authors perform data extraction in duplicate?	Y	N	Y
**7.** Did the review authors provide a list of excluded studies and justify the exclusions?	N	N	N
**8.** Did the review authors describe the included studies in adequate detail?	Y	Y	PY
**9.** Did the review authors use a satisfactory technique for assessing the risk of bias (RoB) in individual studies that were included in the review?	Y	Y	Y
**10.** Did the review authors report on the sources of funding for the studies included in the review?	N	N	N
**11.** If meta-analysis was performed did the review authors use appropriate methods for statistical combination of results?	Y	Y	Y
**12.** If meta-analysis was performed, did the review authors assess the potential impact of RoB in individual studies on the results of the meta-analysis or other evidence synthesis?	Y	Y	Y
**13.** Did the review authors account for RoB in individual studies when interpreting/discussing the results of the review?	Y	Y	Y
**14.** Did the review authors provide a satisfactory explanation for, and discussion of, any heterogeneity observed I the results of the review?	Y	N	N
**15.** If they performed quantitative synthesis did the review authors carry out an adequate investigation of publication bias (small study bias) and discuss its likely impact on the results of the review?	N	N	N

**16.** Did the review authors report any potential sources of conflict of interest, including any funding they received for conducting the review?	N	Y	Y

Abbreviations: N=no; PICO=Population, Intervention, Comparison, Outcome; PY=partial yes; Y=yes
